# Ocrelizumab Discontinuation *vs*. Continuation After Safety Events: Comparative Insights from MSBase

**DOI:** 10.2174/011570159X448901251228053318

**Published:** 2026-03-24

**Authors:** Aurora Zanghì, Tim Spelman, Marzena Fabis-Pedrini, William M. Carroll, Helmut Butzkueven, Allan G. Kermode, Jeannette Lechner-Scott, Anneke van der Walt, Emanuele D’Amico, the MSBase Study Group

**Affiliations:** 1 Department of Medical and Surgical Sciences, University of Foggia, Foggia, Italy;; 2 Department of Clinical Neuroscience, Karolinska Institute, Stockholm, Sweden;; 3 Perron Institute for Neurological and Translational Science, University of Western Australia, Nedlands, Australia;; 4 Personalised Medicine Centre, Health Futures Institute, Murdoch University, Murdoch, Western Australia, Australia;; 5 Perron Institute for Neurological and Translational Science, Sir Charles Gairdner Hospital, QEIIMC, The University of Western Australia, Perth, Australia;; 6 Department of Neurology, The Alfred Hospital, Melbourne, Australia;; 7 Department of Neuroscience, School of Translational Medicine, Monash University, Melbourne, Australia;; 8 Hunter Medical Research Institute, University Newcastle, Newcastle, Australia;; 9 Hunter New England Health, John Hunter Hospital, NSW, Australia

**Keywords:** Ocrelizumab, safety profile, discontinuation, real-world study, multiple sclerosis, high efficacy therapy

## Abstract

**Introduction:**

Ocrelizumab (OCR), a CD20+ B-cell depleting monoclonal antibody, is a highly effective therapy for Multiple Sclerosis (MS). However, safety concerns may lead to treatment discontinuation, raising questions about the clinical consequences of such decisions.

**Materials and Methods:**

This propensity score-matched study utilized data from the MSBase registry to compare outcomes between patients discontinuing OCR due to safety concerns ("Switchers") and those continuing treatment ("Continuers"). Matching was performed using inverse probability of treatment weighting (IPTW) to balance treatment duration and baseline characteristics. Primary outcomes included annualized relapse rate (ARR), time to first relapse, 24-48 weeks confirmed disability worsening (CDW), and progression independent of relapse activity (PIRA).

**Results:**

From an initial cohort of 310 Switchers and 1,315 Continuers, 66 patients who experienced at least one safety event and switched from OCR were matched with 66 Continuers. PS-IPTW analyses revealed higher ARR in Switchers (0.08, 95% CI: 0.05-0.14) *versus* Continuers (0.038, 95% CI: 0.02-0.07; *p=* 0.040). Time to first relapse showed no significant difference (HR=2.23, 95% CI: 0.68-7.28; *p=* 0.183). Trends toward increased CDW24 weeks risk (PS-IPTW HR=2.11, 95% CI: 0.93-4.75; *p=* 0.073), while no significant difference was found at 48 weeks (HR=1.81, 95% CI: 0.83-3.30; *p=* 0.201). PIRA risk showed a trend toward an increase in Switchers (HR=2.45, 95% CI: 0.95-6.29; *p=* 0.063).

**Discussion:**

In this propensity-score-matched analysis, OCR discontinuation due to safety concerns was associated with increased relapse activity and trends toward greater disability progression. These findings highlight the importance of maintaining therapeutic intensity and systematic monitoring during treatment transitions to mitigate safety risks.

**Conclusion:**

Further research is needed to develop strategies for effectively managing adverse events to optimize patient outcomes.

## 1 INTRODUCTION

The therapeutic landscape for Multiple Sclerosis (MS) has expanded significantly, with disease-modifying therapies (DMTs) offering distinct mechanisms of action and well-characterized safety profiles [1, 2]. Ocrelizumab (OCR), a humanized monoclonal antibody targeting CD20+ B cells, has demonstrated efficacy in clinical trial data and real-world evidence studies, providing a robust framework for evaluating its benefit-risk profile [3, 4].

Safety considerations remain a key factor in therapeutic decision-making, particularly during the initiation of treatment, as they influence both patient and clinician preferences [5, 6]. This is especially relevant for OCR, where concerns such as infusion-related reactions and infection risks may impact its adoption despite its established efficacy [7]. Addressing these considerations through clear communication and patient education is essential to support informed decision-making and optimize treatment outcomes.

Clinical trials, including OPERA I, OPERA II, and ORATORIO, have demonstrated OCR's efficacy in reducing relapse rates, slowing disability progression, and decreasing neuroradiological activity [7, 8]. However, as with any immunomodulatory therapy, long-term safety remains a critical consideration. While controlled trials provide initial safety data, real-world evidence is essential for understanding the drug's safety profile across diverse patient populations and clinical settings.

A post-hoc analysis of pooled data from 6,155 patients across 13 clinical trials suggests that continuous long-term treatment with OCR is associated with a manageable infection risk profile [9]. Optimizing disease control and addressing modifiable risk factors may further reduce the risk of infections.

The MSBase registry, a large international observational cohort study, has provided valuable insights into the real-world long-term management of MS therapy [10].

However, a significant gap remains regarding long-term safety and the implications of switching therapies due to safety concerns. The decision to switch from OCR to alternative therapies is complex and involves balancing efficacy, safety, and individual patient factors. Current literature lacks comprehensive analyses comparing the clinical outcomes of patients who switch from OCR due to safety concerns with those who continue treatment.

Our study aims to address this knowledge gap by leveraging data from the MSBase registry. Specifically, we will compare clinical outcomes - including annualized relapse rate (ARR), time to first relapse, Confirmed Disability Worsening (CDW) at 24 and 48 weeks, Progression Independent of Relapse Activity (PIRA)- between patients who switched from OCR due to safety concerns and those who continued treatment despite safety events using a propensity score (PS) matching approach.

## 2 MATERIALS AND METHODS

### 2.1 Study Design and Setting

A multi-centre, longitudinal, observational retrospective cohort study conducted using patient-level data prospectively collected from participants in the MSBase registry. All participant centres provide consent to enrolment in MSBase. Ethics approval for the MSBase registry was granted by the Alfred Health Human Research and Ethics Committee and approval or exemption from the research ethics committee at each participating MSBase referral center (MSBASE 2024-001). Written informed consent was obtained from all enrolled patients in accordance with the declaration of Helsinki. This study followed the Strengthening the Reporting of Observational Studies in Epidemiology (STROBE) reporting guidelines and Gabrielle Simmoneau Guidelines [11, 12]. Patient data were recorded during routine clinic visits at participating centres *via* the locally installed iMed or MDS MSBase data entry systems undergoes a rigorous quality assurance procedure.

### 2.2 Participants

Inclusion criteria were: (A) a diagnosis of relapsing remitting MS (RRMS) according to revised McDonald criteria [13], (B) age >18 years, (C) Expanded Disability Status Scale (EDSS) recorded within 12 months before switching date from OCR (D) the number of treatment relapses on OCR, (E) a minimum of one year of exposure to OCR (the initial split-dose and the second dose of OCR), and (F) a minimum prospectively recorded follow-up: 12 months before the switch and at least two post-baseline disability scores recorded ≥6 months apart (baseline is defined as the day of switching from OCR).

We excluded patients with a primary or secondary progressive MS course at the first evaluation. EDSS scores recorded within 30 days of a relapse were excluded.

Patients were then divided into two groups based on the clinical decision following the occurrence of safety events: the 'Switchers' group (those who switched from OCR) and the 'Continuers' group (those who continued OCR).

Variables included in the dataset were: date of birth, sex, date of disease onset, date of disease course changes, dates of relapses, dates and values of EDSS evaluations, date and type of safety events recorded, and the start and end dates and names of all administered DMTs.

### 2.3 Outcomes and Assessments

Study outcomes included relapse, assessed as ARR and time to CDW, defined as an increase of ⩾ 0.5 point from a baseline EDSS score of >5.5, ⩾ 1.0 point for those with a baseline EDSS score between 1.0 and 5.5, inclusive, and ⩾1.5 points for those with a baseline EDSS score of 0, confirmed after ⩾ 24 weeks and 48 weeks.

Progression Independent of Relapse Activity (PIRA) was defined as an episode of CDW in the absence of relapse during the 90 days prior or 24-weeks following the observed EDSS increase.

All outcomes were calculated from the switch event closely to the safety event, the 'Switchers' cohort, and after the safety event for the 'Continuers' cohort.

Occurring adverse events (AE) were prospectively coded according to the standardized and recognized medical dictionary for regulatory activities (MedDRA) [14]. Outcome and severity of AE were documented. Concomitant medical conditions/comordities occurring for the first time during the treatment were also included.

### 2.4 Statistical Analysis

Categorical variables for the 'Switchers' and 'Continuers' groups were summarised using frequency and percentage. Continuous variables were summarised using mean and standard deviation (SD); or median and interquartile range (IQR) as appropriate. Time-to-event outcomes were summarised and visualised using Kaplan-Meier estimates.

'Switchers' were matched on a 1:1 basis to 'Continuers' based on treatment duration of OCR at the index date. Propensity score (PS) weighting was then used to adjust for imbalances in the distribution of confounders between the matched 'Switchers' and 'Continuers' groups.

The propensity score weight was derived using a logistic regression where group status ('Switchers' *vs*. 'Continuers') was defined as the dependent outcome variable, and age, sex, country, disease duration, time since diagnosis, baseline EDSS, and the number of pre-OCR disease modifying therapies (DMTs) were defined as the independent explanatory confounder variables. Weighted standardised differences were used to assess the balance of confounders between 'Switchers' and 'Continuers' groups.

PS inverse probability of treatment weighting (PS-IPTW) negative binomial regression was used to compare the total count of relapse between groups.

PS-IPTW marginal Cox regression was used to compare time to first relapse, CDW at 24 and 48 weeks, and PIRA between the 'Switchers' and 'Continuers' groups. A subgroup analysis limiting the analysis to 'Switchers' who switched to high efficacy treatment (alemtuzumab, cladribine, mitoxantrone, ofatumumab, or rituximab) and the matched 'Continuers' was undertaken.

A sensitivity analysis, further adjusting for time between OCR discontinuation and subsequent DMT start, was also undertaken.

For all analyses, *p*<0.05 was considered significant. All analyses were undertaken using Stata version 18 (StataCorp. 2023. Stata Statistical Software: Release 18. College Station, TX: StataCorp LLC).

## 3 RESULTS

From a total cohort of 107,748 patients in MSBase at the extract date, 1,315 patients were enrolled in the Continuers cohort with a safety event, and 310 were enrolled in the Switchers cohort with a safety event (Table **1**). After 1:1 matching, 66 patients were included in the following analyses. Baseline characteristics for the overall and 1:1 matched investigated cohorts are shown in Table **2**.

Before matching, demographic characteristics showed imbalances between groups, particularly in geographic distribution (Australian patients: 55.2% *vs.* 46.7%), age (standardized difference -0.164), and gender (standardized difference -0.157). After matching and PS weighting based on key variables including age, sex, country, disease duration, time since diagnosis, EDSS, and pre-OCR DMT count, these imbalances were reduced (weighted standardized differences -0.098 to -0.122), demonstrating adequate balance between the matched cohorts.

The mean time on OCR for Switchers was 2.7 ± 1.2 years, whilst for the Continuers was 4.4 ± 1.2 years. The mean wash-out time for Switchers was 180 days (IQR 20-281).

The type and distribution of safety events according to the matched cohort are shown in Table **3**.

The most frequently reported safety AEs in the matched Switchers cohort were infections (24/66, 36.4%), followed by cancers (7/66, 10.6%) and other concomitant medical conditions.

### 3.1 Clinical outcomes

#### 3.1.1 Annualized Relapse Rate, Relapse Count, and Time to First Relapse

ARR was higher in Switchers than Continuers: 0.08 (95% CI 0.05-0.14) *vs.* 0.038 (0.02, 0.07), *p=*0.040. A total number of 26 relapses were recorded (15 in Switchers and 11 in Continuers), within a cumulative follow-up of 180.3 months and 281.4 months, respectively. The PS-IPTW negative binomial regression model for relapse count was associated with a higher risk for Switchers than Continuers: unadjusted RR 2.40 (95% CI 0.88, 6.58) 0.088; adjusted RR 2.62 (95% CI 0.96-7.15), although this difference was marginally non-significant (*p=*0.060). The subgroup switching to high efficacy DMTs (n=44) did not differ for total count of relapse, unadjusted RR 1.66 (95% CI 0.55, 5.03), *p=*0.368; adjusted RR 1.77 (95% CI 0.62, 5.07), *p=*0.284.

**Fig. (1):**
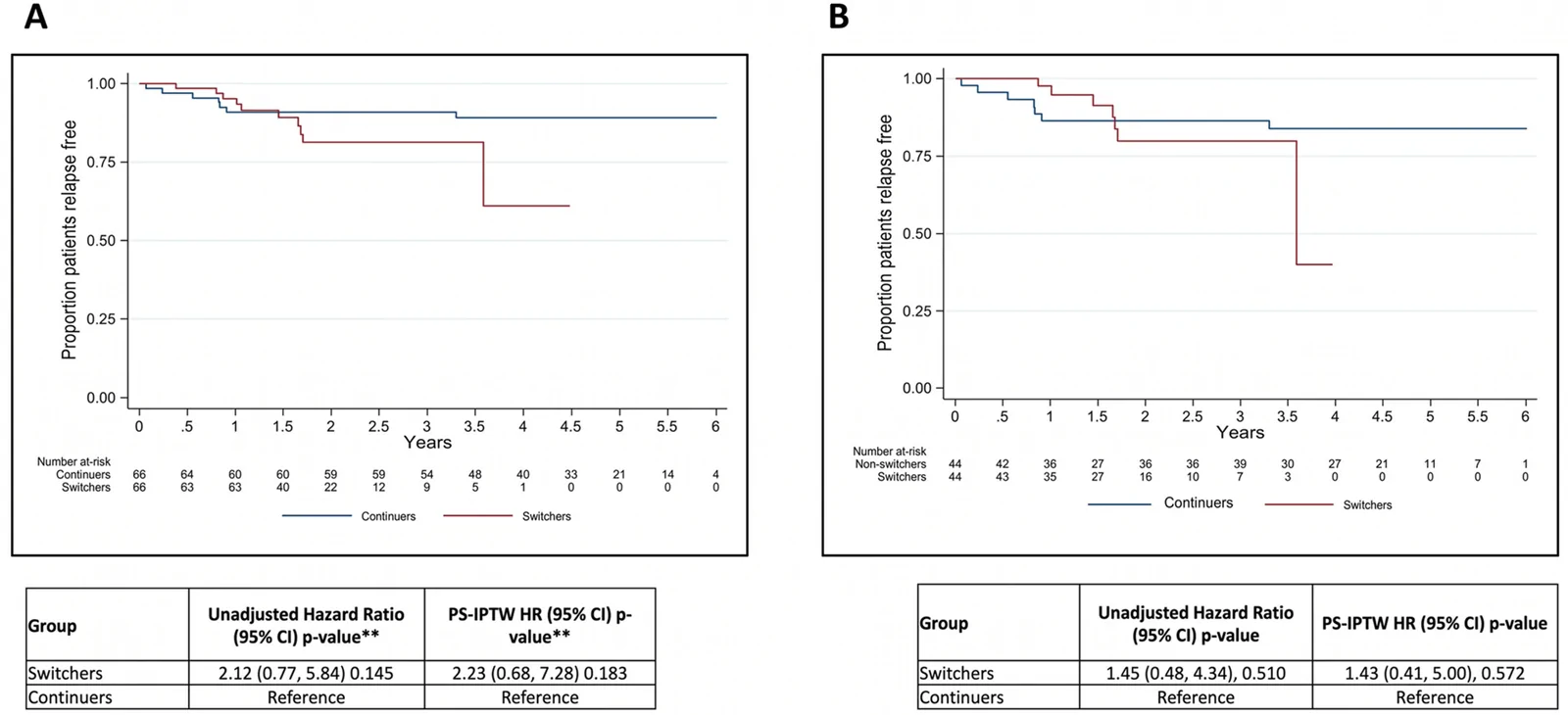
Time to first relapse: continuers *vs.* switchers. (**A**). All cohort, (**B**) Subgroup switching to high efficacy DMTs, *Marginal Cox model, **Propensity score - Inverse Probability of Treatment Weighting (PS-IPTW) Marginal Cox model. **Abbreviations**: DMTs disease modifying therapies; CI, confidence interval; HR, Hazard ratio.

Time to first relapse did not show differences between the two groups in the whole cohort: adjusted HR 2.23 (0.68, 7.28), *p=* 0.183 (Fig. **1A**), and also in the subgroup switching to high-efficacy DMTs (Fig. **1B**).

#### 3.1.2 Confirmed Disability Worsening at 24 and 48 Weeks

**Fig. (2):**
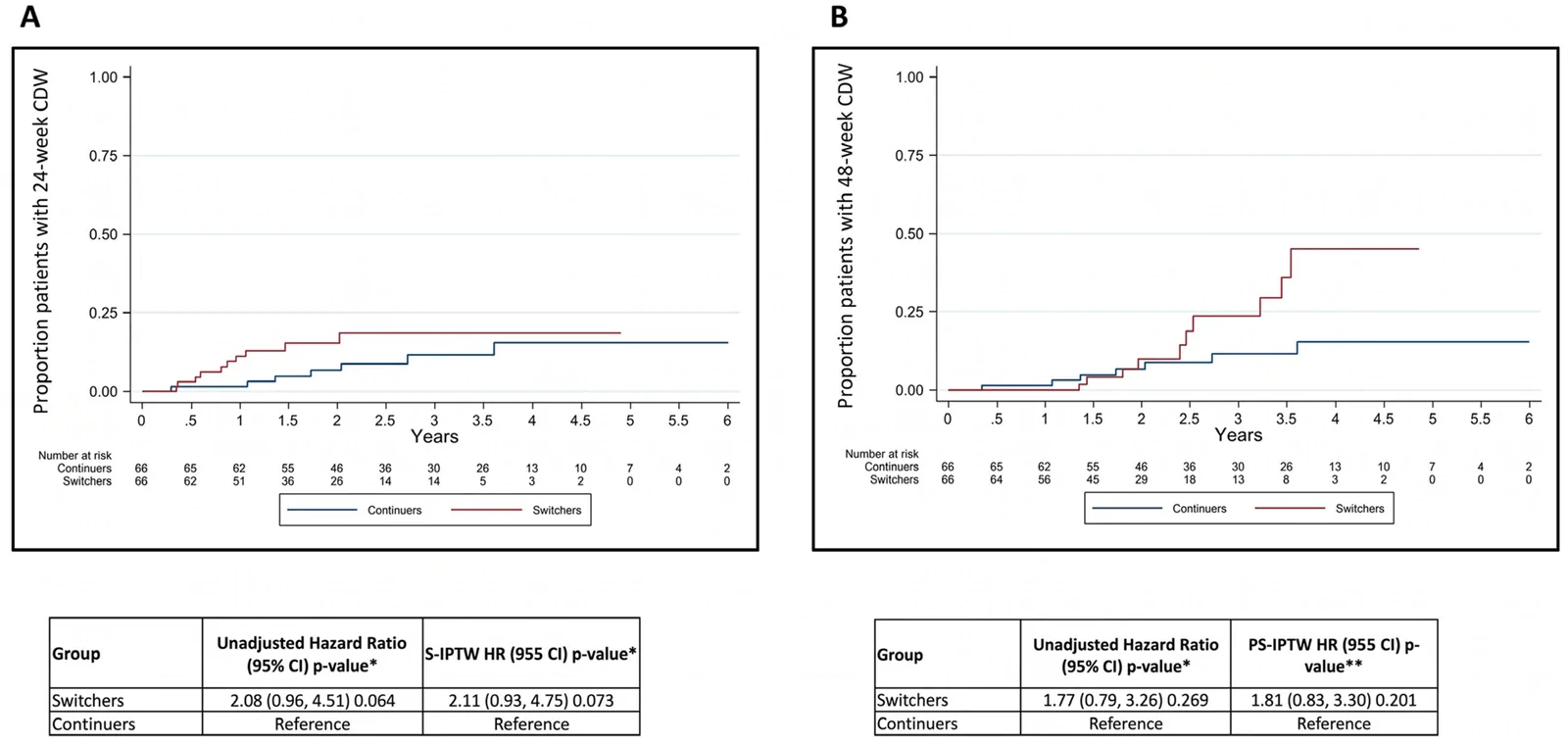
Confirmed Disability Worsening at 24 and 48 weeks: continuers *vs.* switchers. (**A**) 24 weeks-CDW-all cohort, (**B**) 48 weeks-CDW-all cohort. *Marginal Cox model, **Propensity score - Inverse Probabiltiy of Treatment Weighting (PS-IPTW) Marginal Cox model. **Abbreviations**: CDW, confirmed disability worsening; CI, confidence interval; DMTs disease modifying therapies; HR, hazard ratio.

**Fig. (3):**
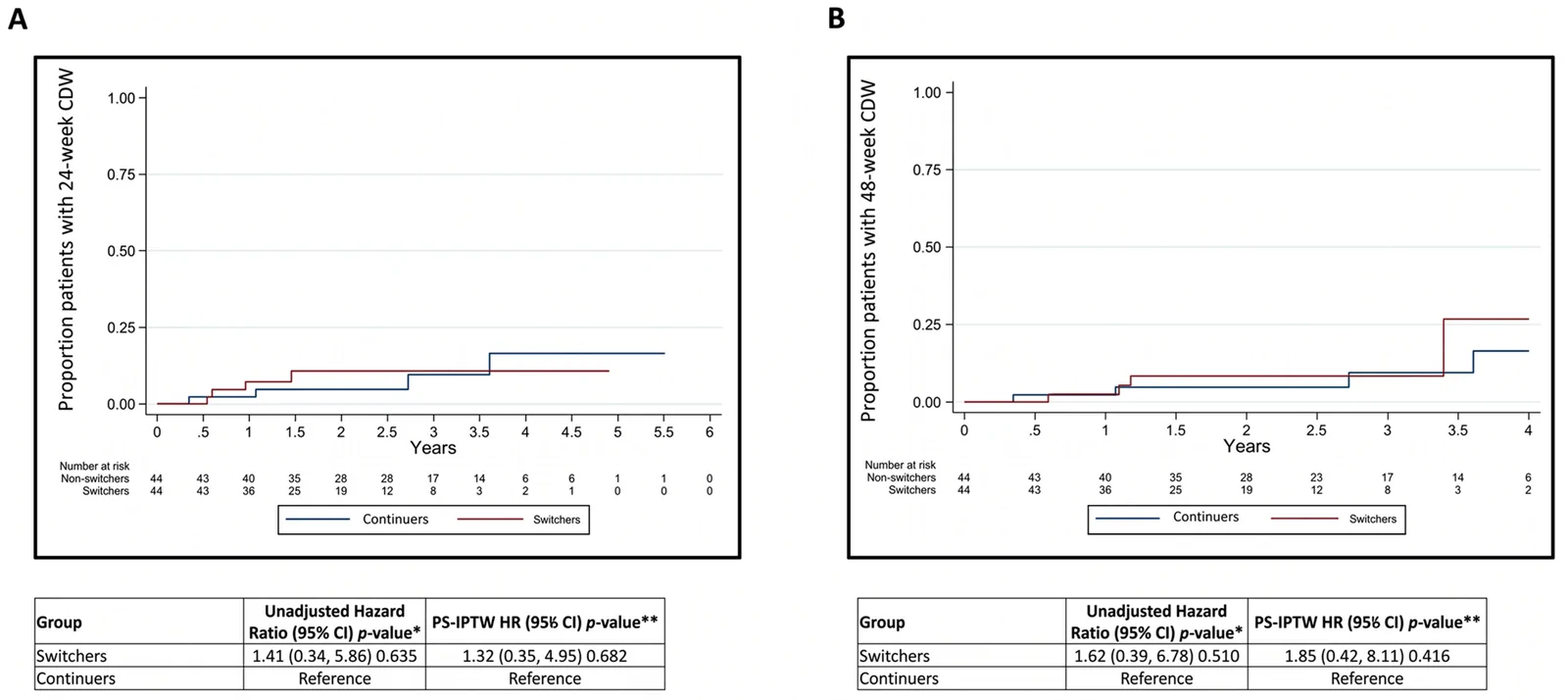
Confirmed Disability Worsening at 24 and 48 weeks subgroup analysis: continuers *vs.* switchers. (**A**) 24 weeks-CDW- subgroup switching to high efficacy DMTs, (**B**) 48 weeks-CDW- subgroup switching to high efficacy DMTs. *Marginal Cox model, **Propensity score - Inverse Probabiltiy of Treatment Weighting (PS-IPTW) Marginal Cox model. **Abbreviations**: CDW, confirmed disability worsening; CI, confidence interval; DMTs disease modifying therapies; HR, hazard ratio.

During the investigated follow-up, ten CDW-24 weeks events occurred in Switchers and 7 in Continuers. Time to CDW-24 weeks showed a trend toward higher risk for Switchers than Continuers (PS-IPTW HR 2.11 [95% CI 0.93-4.75], *p*-value=0.073) while time to CDW 48 weeks did not show differences between the two groups (PS-IPTW HR 1.81 [95% CI 0. 83-3.30], *p*-value=0.201 (Figs. **2A** and **B**). No difference in time to CDW 24 and 48 weeks was observed when considering the subgroup switching to high efficacy DMTs (Figs. **3A** and **B**).

#### 3.1.3 Progression Independent of Relapse Activity

A total number of 16 PIRA events were recorded, 10 in Switchers and 6 in Continuers.

**Fig. (4):**
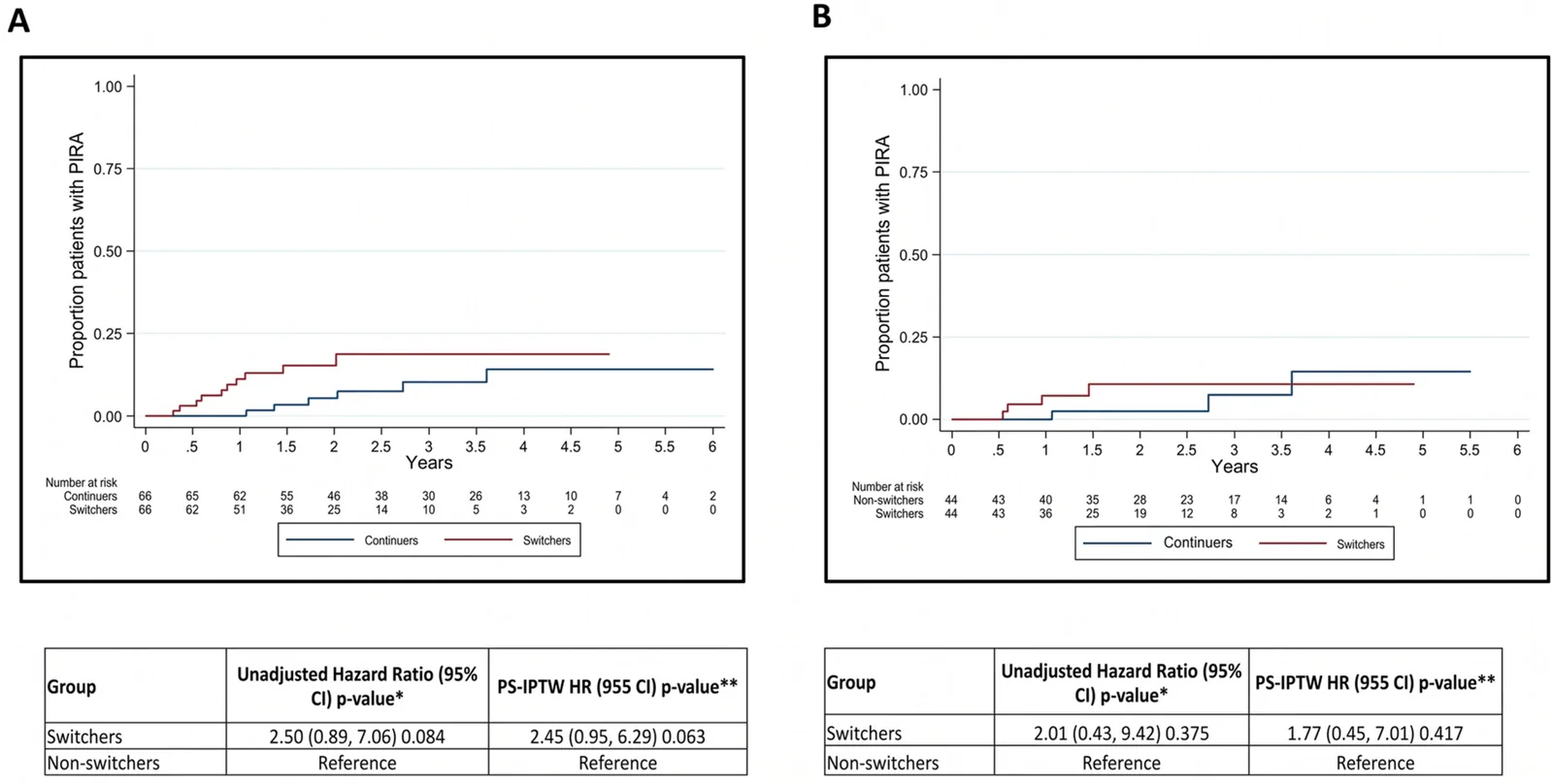
Progression independent of relapse activity: continuers *vs.* switchers. (**A**) All cohort, (**B**) Subgroup switching to high efficacy DMTs, *Marginal Cox model **Propensity score - Inverse Probability of Treatment Weighting (PS-IPTW), **Abbreviations**: DMTs disease modifying therapies; CI, confidence interval; HR, Hazard ratio.

The IPTW-PS Cox marginal model showed a higher, albeit non-significant, event rate in the Switchers: HR 2.45 (95% CI 0.95, 6.29) 0.063 (Fig. **4A**). No differences were observed in patients switching to high efficacy DMTs (Fig. **4B**).

### 3.2 Sensitivity Analysis

The sensitivity analysis was performed, adjusting for time between OCR discontinuation and subsequent switch (for Switchers cohort) in the matched cohort.

The PS-IPTW negative binomial regression model for relapse count was associated with a higher risk for Switchers than Continuers: adjusted RR 2.62 (95% CI 1.00-6.51), although this association was marginally non-significant (*p=*0.051). Time to first relapse did not differ between two cohort: PS-IPTW HR 2.11 (95% CI 0.67, 6.65), *p*-value=0.202.

Time to CDW 24 and 48 weeks did not differ between the two groups: PS-IPTW HR 2.05 (95% CI 0.81, 5.20), *p=*0.129 (24 weeks) and PS-IPTW HR 1.75 (95%CI 0.77, 2.23).

Whilst Switchers were associated with a higher risk of PIRA (HR 2.41 (95% CI 0.93, 6.27)), although this association was non-significant (*p=*0.070).

## 4 DISCUSSION

The analysis of patients discontinuing OCR due to safety events revealed findings that require careful interpretation. While initial evaluations suggested differences in ARR and progression metrics, the statistical evidence for the latter demands thoughtful consideration. The observed p-values in the CDW-24 weeks and PIRA analyses, although not meeting conventional significance thresholds, warrant attention within the context of this well-matched cohort.

These borderline p-values, particularly in the paired analysis, indicate a trend that becomes especially relevant when considering therapeutic transitions. Interestingly, this statistical pattern disappeared when examining the subgroup transitioning to high-efficacy alternatives, potentially suggesting that maintaining therapeutic intensity may help preserve clinical stability. Furthermore, the sensitivity analysis, adjusted for the washout period, confirmed the results obtained in the whole matched cohort, except for CDW-24 weeks.

While the presence of safety concerns undoubtedly compels clinicians to thoroughly evaluate the risk-benefit profile, the results highlight the critical importance of maintaining high-efficacy therapies.

The higher observed, albeit marginally non-significant, ARR and the relapse count after adjustment in switchers align with substantial evidence from pivotal trials and real-world studies showcasing OCR's effectiveness in managing disease activity.

Based on the findings, the de-escalating protocols should be weighed cautiously. Previous studies have suggested that switching from OCRE to low-moderate efficacy DMTs may be an effective treatment option for people with RRMS who are clinically stable but may need to switch for reasons unrelated to effectiveness, or in MS patients older than 55 years [15]. However, despite several small studies, a definite conclusion about treatment de-escalation does not exist [16].

After discontinuation of anti-CD20 therapy, peripheral B-cell reconstitution may lead to transient immune reactivation, characterized by repopulation of naïve and memory B-cell subsets and restoration of antigen-presenting function [17, 18]. This immunological rebound can potentially contribute to increased inflammatory activity in susceptible patients [18]. Maintaining continuous high-efficacy therapy may therefore help minimize periods of immune reactivation and sustain long-term disease stability. CDW-24 weeks was higher in, and it is in accordance with the previous study [19]. The OPERA I/II extension studies revealed that sustained OCR treatment reduced 24-week confirmed disability progression by 40% compared to interferon β-1a, maintaining its effects over 6.5 years [19].

Timing of switch after anti-CD20 agents should also warrant further investigation, as, although we cannot infer on this, the significant association CDW-24 weeks was not maintained in the sensitivity analysis after adjustment for washout [3, 20].

Our analysis identified a trend toward increased PIRA risk (*p=*0.060) among patients who switched treatments. While these results did not reach statistical significance, they merit consideration in the context of treatment decisions.

The mechanism behind OCR's influence on PIRA operates through multiple pathways [21]. While its primary action involves peripheral B-cell depletion, OCR also reduces key cerebrospinal fluid biomarkers of neurodegeneration, including neurofilament light chain, consistent with its effects on acute disease activity and disability progression [22]. Persistently elevated neurofilament light chain levels have also been observed in a subgroup of persons under ocrelizumab treatment, demonstrating a potential clinical utility as a predictive biomarker of increased risk for clinical progression [22].

Similar principles of structured risk management have been successfully implemented for other high-efficacy therapies in MS. For instance, the monitoring and stratification strategies developed for natalizumab-associated progressive multifocal leukoencephalopathy have demonstrated how systematic evaluation of individual risk factors, such as JC virus serostatus, treatment duration, and prior immunosuppressant exposure, can mitigate severe adverse outcomes [23, 24]. Translating comparable proactive approaches to ocrelizumab-treated patients could further enhance long-term safety without compromising therapeutic efficacy.

The safety profile leading to OCR discontinuation was mostly related to severe infections emerging, followed by infections of varying severity and cancers. This distribution provides critical insights for clinical decision-making and patient risk stratification, informing the development of tailored monitoring protocols.

While safety concerns warrant attention, OCR's safety profile remains manageable with appropriate monitoring, suggesting that treatment benefits often outweigh potential risks. This underscores the importance of systematic risk assessment, as treatment discontinuation may be associated with disease progression.

The findings support the implementation of a standardized approach to adverse event classification within MS care.

Preventing irreversible progression remains a major therapeutic priority, particularly as accumulating evidence supports the value of early, individualized treatment strategies to delay disability accrual in progressive forms of MS [25, 26].

The classification of side effects as more or less severe is crucial, as the price of discontinuing treatment may be a worsening of the disease. In the context of MS, long-term management and the involvement of a multidisciplinary team are essential. Specialists such as infectious disease experts and haematologists play a critical role in comprehensive MS care. The concept of an MS care unit underscores the importance of specialized training and collaboration among healthcare professionals. Ensuring that neurologists and other specialists are well-equipped to manage the complexities of MS is vital for optimizing patient outcomes and also healthcare resources [27, 28]. In MS care, united expertise is essential for preserving brain health.

This study has several strengths. First, its real-world setting provides insights into OCR's effectiveness and safety in routine clinical practice, enhancing external validity. Unlike controlled trials, real-world studies capture the complexities of patient management, making results more applicable to everyday clinical scenarios.

Second, the use of propensity score matching represents a significant methodological strength. By achieving well-balanced cohorts (n=66 per group) with weighted standardized differences below 0.122 for all baseline characteristics, the study minimizes selection bias and confounding variables. This rigorous matching process ensures fair comparison between Switchers and Continuers, lending credibility to the observed trends.

## 5 LIMITATIONS

Despite its strengths, several limitations warrant consideration. The primary limitation stems from the relatively small sample size of matched cohorts (n=66 per group), which reduces statistical power and limits the ability to detect significant differences, particularly for less frequent outcomes. This constraint is especially relevant given the borderline statistical findings (*p=*0.06-0.07) observed in key analyses. Additionally, unmeasured or residual confounding remains possible as the study's aim assumed that the safety event in the switchers was the most suitable reason for OCR discontinuation and also the challenge of safety event *vs.* underlying condition/comorbidity, caution in interpreting the occurrence of safety event and association with the treatment.

The observational nature introduces potential unmeasured confounders, despite rigorous propensity score matching. While matching minimizes bias, it cannot account for all variables influencing treatment outcomes, such as differences in monitoring intensity or adherence to treatment protocols.

Variable follow-up durations between Switchers and Continuers may introduce temporal bias, potentially affecting outcome assessment. This variability influences trend interpretation, particularly regarding disease progression and safety events, and MRI information was not available.

## CONCLUSION

The heterogeneity of safety events leading to OCR discontinuation presents a challenge for generalizability. The small subgroups created by this heterogeneity limit conclusions about specific safety scenarios, underscoring the need for larger validation studies.

Treatment modification decisions require a systematic evaluation of disease reactivation risk in relation to individual safety concerns. When OCR discontinuation becomes necessary, maintaining therapeutic intensity through alternative high-efficacy treatments may help preserve disease control. Systematic monitoring of clinical and radiological disease activity, particularly PIRA markers, remains essential during treatment transitions.

Future investigations should address current methodological limitations while expanding the evidence base for treatment transitions in MS. Larger cohort studies with extended follow-up would enhance statistical power and enable robust subgroup analyses, particularly for less frequent adverse events.

In conclusion, our study highlights the importance of balancing safety considerations with the therapeutic benefits of continued OCR treatment. Further research should focus on developing risk-mitigation and monitoring strategies to optimize patient outcomes.

## Figures and Tables

**Table 1:** Inclusion criteria and cohort selection.

**Inclusion Critera**	**n.**
**Set 1-all**	
All patients in MSBase at extract date*	107,748
Excluding opt-out centres	106,866
Started OCR	9,673
Confirmed MS diagnosis	9,208
**Set -Continuers**	
Confirmed MS diagnosis	9,208
Minimum one year on OCR	6,938
Minimum two EDSS scores on OCR (post safety event)	4,161
Recorded an on OCR safety event	**1,315**
**Set-Switchers**	
Discontinued OCR	703
EDSS recorded 12 months before a switch from OCR (at least 1)	531
Minimum one year on OCR	603
Minimum 12 months pre-switch follow-up	671
Minimum two EDSS scores post switch	393
Satisfy all inclusion criteria	**310**

**Note**: *MSBase extact date: 1^st^ April 2024.

**Abbreviations**: EDSS, Expanded Disability Status Scale; MS, Multiple Sclerosis; n., number; OCR, ocrelizumab.

**Table 2:** Baseline characteristics of the enrolled cohort.

-	-	**Unmatched Switch Cohort**	**Matched Switch Cohort**	**Unmatched Continuers Comparator Cohort**	**Matched Continuers Comparator Cohort**	**-**	-
**Characteristic at Index Date**	**Category/Metric**	**n=310**	**n=66**	**n=1315**	Matched Cohort - (n=66)***	**Standardised Difference**	Weighted Standardised Difference****
Age (years)	Mean (SD)	46.12 (11.1)	46.28 (12.3)	**	48.2 (11.2)	-0,164	-0,098
	Median (IQR)	45.7 (37.3, 54.5)	44.81 (36.4, 56.2)	**	47.8 (40.7, 54.6)		
Sex - n (%)	Female	217 (70.0)	46 (69.7)	949 (72.2)	50 (75.8)	-0,135	-0,122
	Male	93 (30.0)	20 (30.3)	366 (27.8)	16 (24.2)		
Country - n (%)	Australia	171 (55.2)	48 (72.7)	614 (46.7)	40 (60.6)	-0,157	-0,122
	Turkey	46 (14.8)	8 (12.1)	170 (12.9)	6 (9.1)		
	Czechia	36 (11.6)	3 (4.6)	198 (15.1)	5 (7.6)		
	Canada	27 (8.7)	2 (3.0)	35 (2.7)	3 (4.6)		
	Kuwait	7 (2.3)	2 (3.0)	41 (3.1)	1 (1.5)		
	Belgium	6 (1.9)	1 (1.5)	49 (3.7)	1 (1.5)		
	Italy	6 (1.9)	0 (0.0)	42 (3.2)	0 (0.0)		
	Spain	3 (1.0)	0 (0.0)	36 (2.7)	4 (6.1)		
	Croatia	2 (0.7)	1 (1.5)	48 (3.7)	3 (4.6)		
	Switzerland	1 (0.3)	1 (1.5)	32 (2.4)	0 (0.0)		
	Colombia	1 (0.3)	0 (0.0)	4 (0.3)	1 (1.5)		
	Estonia	1 (0.3)	0 (0.0)	0 (0.0)	0 (0.0)		
	Iran	1 (0.3)	0 (0.0)	2 (0.2)	0 (0.0)		
	Oman	1 (0.3)	0 (0.0)	0 (0.0)	0 (0.0)		
	Saudi Arabia	1 (0.3)	0 (0.0)	5 (0.4)	0 (0.0)		
	Brazil	-	0 (0.0)	3 (0.2)	1 (1.5)		
	Egypt	-	0 (0.0)	1 (0.1)	0 (0.0)		
	Greece	-	0 (0.0)	3 (0.2)	0 (0.0)		
	Lebanon	-	0 (0.0)	7 (0.5)	0 (0.0)		
	Netherlands	-	0 (0.0)	8 (0.6)	1 (1.5)		
	New Zealand	-	0 (0.0)	15 (1.1)	0 (0.0)		
	Slovakia	-	0 (0.0)	1 (0.1)	0 (0.0)		
	Tunisia	-	0 (0.0)	1 (0.1)	0 (0.0)		
Disease duration (time since first symptoms) (years)	Mean (SD)	13.8 (8.8)	14.4 (9.3)	**	16.5 (10.7)	-0,204	-0,148
	Median (IQR)	12.67 (7.0, 19.4)	12.7 (6.6, 20.9)	**	16.0 (6.8, 21.9)		
Time since diagnosis (years)	Mean (SD)	11.1 (7.7)	11.5 (8.8)	**	13.28 (9.56)	-0,200	-0,147
	Median (IQR)	9.5 (4.6, 15.6)	8.8 (3.5, 16.8)	**	12.6 (4.4, 18.9)		
EDSS at index date	Median (IQR)	-	4 (2, 6)	-	3.25 (1.5, 6)	-0,006	-0,021
Count of pre-OCR DMTs	Mean (SD)	-	2.5 (2.3)	-	2.3 (2.9)	0,075	0,039
	Median (IQR)	-	2 (0, 4)	-	1 (0, 3)		
Last EDSS in the 12 months prior to OCR end	Mean (SD)	4.21 (2.10)	3.89 (2.20)	N/A	N/A	N/A	N/A
	Median (IQR)	4.5 (2.5, 6)	4 (2, 6)	N/A	N/A		
Post OCR switch DMT - n (%)	Ofatumumab	-	18 (27.3)	N/A	N/A	N/A	N/A
	Cladribine	-	14 (21.2)	N/A	N/A		
	Natalizumab	-	11 (16.7)	N/A	N/A		
	Siponimod	-	11 (16.7)	N/A	N/A		
	DMF	-	4 (6.1)	N/A	N/A		
	Fingolimod	-	3 (4.6)	N/A	N/A		
	Glatiramer acetate	-	3 (4.6)	N/A	N/A		
	Rituximab	-	1 (1.5)	N/A	N/A		
	Teriflunomide	-	1 (1.5)	N/A	N/A		

**Dependent on matched baseline date.

***Matched on time on OCR at first safety event (non-switcher arm) or time until switch (switcher arm).

****Propensity score weight derived on age, sex, country, disease duration, time since diagnosis, EDSS and pre-OCR DMT count.

Index date: date of end OCR treatment post safety event (switchers), date of first safety event (non-switchers).

**Table 3:** Adverse events in the switchers cohort (A) and in the continuers cohort (B).

**(A)**	
**Events Recorded According to MedDRA**	**Switchers n.**
Asthma	1
Cardiac arrhythmia	2
Cervical dysplasia	1
Colitis	2
Chron disease	2
Flu-like symptoms	5
Headache	3
Hepatic disorders	1
Hepatitis	1
Impetigo	1
Irritable bowel disease	1
Metabolic diseases	5
Nausea, vomiting, diarrhea	2
Neutropenia	3
Otitis externa	1
Pericarditis	1
Pseudomonas infection	1
Psychiatric diseases	5
Seizures/convulsions	1
Severe upper respiratory tract infection	5
Urinary tract infection	8
Subclavian artery stenosis	1
Stroke	1
Shingles	2
Urosepsis	1
Other Infections	2
Viral Infection	1
Tyroid carcinoma	1
Breast tumor	1
Malignancy right arm	1
Malignancy back	1
Non melanoma skin cancer face and eye	1
Non melanoma skin cancer lips	1

**Table:** 

**(B)**	
**Events Recorded According to MedDRA**	**Continuers n.**
Acne	1
Acoustic Neuroma	1
Anemia	2
Arthralgia	1
Arthritis	1
Asthma	1
Barrett's oesophagus	1
Bell facialisparalysis	1
Boil by *Methicillin-Resistant Staphylococcus Aureus*	1
Candidiasis	1
Cardiovascular disorders	1
Cellulitis	1
Cervical myelopathy	2
Chlamydia infection	1
Chronic obstructive pulmonary disease	1
Cyotmegalovirus reactivation	1
Coronary artery disease-Angina pectoris	2
COVID-19 infection	1
Colitis	2
Dermatological disorders	1
Eczema	1
Endocrine disorders	1
Epilepsy	1
Severe Fatty liver	1
Febrile illness	10
Gastroenteritis	2
Herpes zoster	1
Influenza	1
Liver test(s) abnormality	1
Migraine	4
Muscle ache	1
Nausea	1
Neutropenia	1
Otitis	1
Vertigo	2
Psychiatric comorbidities	
Psoriasis	1
Rash, swaeting, fever	4
Lower Respiratory Tract infection	2
Shingles	1
Skin lesion (benign)	1
Upper Respiratory Tract infection	2
Urinary Tract Infection	2

## Data Availability

The data that support the findings of this study are available from the corresponding author, (E.D’A.), upon reasonable request.

## LIST OF ABBREVIATIONS

AE: Adverse event
ARR: Annualized Relapse Rate
CD20⁺: Cluster of Differentiation 20 Positive
CDW: Confirmed Disability Worsening
CI: Confidence Interval
DMT: Disease-modifying Therapy
EDSS: Expanded Disability Status Scale
HR: Hazard Ratio
IQR: Interquartile Range
MedDRA: Medical Dictionary for Regulatory Activities
MS: Multiple Sclerosis
OCR: Ocrelizumab
PIRA: Progression Independent of Relapse Activity
PS-IPTW: Propensity Score–inverse Probability of Treatment Weighting
RR: Relative Risk
RRMS: Relapsing-remitting Multiple Sclerosis
SD: Standard Deviation
STROBE: Strengthening the Reporting of Observational Studies in Epidemiology.

## STANDARDS OF REPORTING

STROBE guidelines were followed.

## COLLABORATORS

•  Nevin John, MBBS, PhD, Monash University, Clayton, Australia.

•  Tomas Kalincik, MD, PhD, PGCertBiostat, Neuroimmunology Centre, Department of Neurology, Royal Melbourne Hospital, Melbourne, Australia.

•  Izanne Roos, MBChB, PhD, Neuroimmunology Centre, Department of Neurology, Royal Melbourne Hospital, Melbourne, Australia.

•  Cavit Boz, MD, Karadeniz Technical University, Medical Faculty, Trabzon, Turkey.

•  Serkan Ozakbas, MD, Izmir University of Economics, Medical Point Hospital, Izmir, Turkey.

•  Michael Barnett, MBBS, PhD Brain and Mind Centre, Sydney, Australia.

•  Suzanne Hodgkinson, MBBS, PhD, UNSW, Sydney, Australia.

•  Mario Habek MD, PhD, University Hospital Center Zagreb, Zagreb, Croatia.

•  Katherine Buzzard, MBBS, PhD, Department of Neurosciences, Box Hill Hospital, Box Hill, Australia Olga Skibina, MBBS, PhD, Department of Neurology, Box Hill Hospital, Melbourne, Australia.

•  Pierre Grammond, MD, CISSS Chaudière-Appalache, Levis, Canada.

•  Miroslav Mares, MD, Hospitals of the Pardubice Region, Hospital of Pardubice, Pardubice, Czech Republic.

•  Raed Alroughani, MD, Amiri Hospital, Sharq, Kuwait.

•  Guy Laureys, MD, Universitary Hospital Ghent, Ghent, Belgium.

•  Dana Horakova, MD, PhD, Charles University in Prague and General University Hospital, Prague, Czech Republic.

•  Eva Kubala Havrdova, MD, PhD, Charles University in Prague and General University Hospital, Prague, Czech Republic.

•  Guillermo Izquierdo, MD, Hospital Universitario Virgen Macarena, Sevilla, Spain.

•  Sara Eichau, MD, Hospital Universitario Virgen Macarena, Sevilla, Spain.

•  Pamela McCombe, MD, Royal Brisbane Hospital, Brisbane, Australia.

•  Jennifer Massey, MD, St Vincent's Hospital, Sydney, Australia.

•  Maria Cecilia Aragon de Vecino, Hospital Moinhos de Vento, Multiple Sclerosis Unit, Porto Alegre, Brazil.

•  Alexandre Prat, MD, PhD, CHUM and Universite de Montreal, Montreal, Canada.

•  Marc Girard, MD, CHUM and Universite de Montreal, Montreal, Canada.

•  Pierre Duquette, MD, CHUM and Universite de Montreal, Montreal, Canada.

•  Jiwon Oh, MD, St. Michael's Hospital, Toronto, Canada.

•  Jens Kuhle, MD, University Hospital and University of Basel, Basel, Switzerland.

•  Simón Cárdenas-Robledo, Hospital Universitario Nacional de Colombia Bogota, Colombia.

•  CPavel Hradilek, Assoc. Prof., MD, PhD, University Hospital Ostrava, Ostrava, Czech Republic.

•  Ivana Stetkarova, Prof., MD, PhD, MHA, Third Faculty of Medicine, Charles University and Hospital Kralovske Vinohrady, Prague, Czech Republic.

•  Marek Peterka MD, Faculty of Medicine in Pilsen, Charles University, and University Hospital Pilsen, Pilsen, Czech Republic.

•  Cristina Ramo-Tello, MD, PhD, Hospital Germans Trias i Pujol, Badalona, Spain.

•  Yolanda Blanco, MD, Hospital Clinic de Barcelona, Barcelona, Spain.

•  Bassem Yamout, MD, Harley Street Medical Center, Abu Dhabi-UAE.

•  Samia J. Khoury, MD, American University of Beirut Medical Center, Beirut, Lebanon.

•  Oliver Gerlach, MD, Academic MS Center Zuyd, Department of Neurology, Zuyderland Medical Center, Sittard-Geleen, Netherlands.

## References

[1] Amin M., Hersh C.M. (2023). Updates and advances in multiple sclerosis neurotherapeutics.. Neurodegener. Dis. Manag..

[2] Jakimovski D., Bittner S., Zivadinov R., Morrow S.A., Benedict R.H.B., Zipp F., Weinstock-Guttman B. (2024). Multiple sclerosis.. Lancet.

[3] de Sèze J., Maillart E., Gueguen A., Laplaud D.A., Michel L., Thouvenot E., Zephir H., Zimmer L., Biotti D., Liblau R. (2023). Anti-CD20 therapies in multiple sclerosis: From pathology to the clinic.. Front. Immunol..

[4] Carlson A.K., Amin M., Cohen J.A. (2024). Drugs targeting CD20 in multiple sclerosis: Pharmacology, efficacy, safety, and tolerability.. Drugs.

[5] Stoll S., Costello K., Newsome S.D., Schmidt H., Sullivan A.B., Hendin B. (2024). Insights for healthcare providers on shared decision-making in multiple sclerosis: A narrative review.. Neurol. Ther..

[6] Mahmoudi N., Wattjes M.P. (2024). Treatment monitoring in multiple sclerosis — Efficacy and safety.. Neuroimaging Clin. N Am.

[7] Hauser S.L., Kappos L., Montalban X., Craveiro L., Chognot C., Hughes R., Koendgen H., Pasquarelli N., Pradhan A., Prajapati K., Wolinsky J.S. (2021). Safety of ocrelizumab in patients with relapsing and primary progressive multiple sclerosis.. Neurology.

[8] Vermersch P., Oreja-Guevara C., Siva A., Van Wijmeersch B., Wiendl H., Wuerfel J., Buffels R., Kadner K., Kuenzel T., Comi G. (2022). Efficacy and safety of ocrelizumab in patients with relapsing‐remitting multiple sclerosis with suboptimal response to prior disease‐modifying therapies: A primary analysis from the phase 3b CASTING single‐arm, open‐label trial.. Eur. J. Neurol..

[9] Derfuss T., Bermel R., Lin C.J., Hauser S.L., Kappos L., Vollmer T., Comi G., Giovannoni G., Hartung H.P., Weber M.S., Wang J., Jessop N., Chognot C., Craveiro L., Bar-Or A. (2024). Long-term analysis of infections and associated risk factors in patients with multiple sclerosis treated with ocrelizumab: Pooled analysis of 13 interventional clinical trials.. Ther. Adv. Neurol. Disord..

[10] Butzkueven H., Chapman J., Cristiano E., Grand’Maison F., Hoffmann M., Izquierdo G., Jolley D., Kappos L., Leist T., Pöhlau D., Rivera V., Trojano M., Verheul F., Malkowski J.-P. (2006). MSBase: An international, online registry and platform for collaborative outcomes research in multiple sclerosis.. Mult. Scler..

[11] Simoneau G., Pellegrini F., Debray T.P.A., Rouette J., Muñoz J., Platt R.W., Petkau J., Bohn J., Shen C., de Moor C., Karim M.E. (2022). Recommendations for the use of propensity score methods in multiple sclerosis research.. Mult. Scler..

[12] von Elm E., Altman D.G., Egger M., Pocock S.J., Gøtzsche P.C., Vandenbroucke J.P. (2008). The Strengthening the Reporting of Observational Studies in Epidemiology (STROBE) statement: Guidelines for reporting observational studies.. J. Clin. Epidemiol..

[13] Thompson A.J., Banwell B.L., Barkhof F., Carroll W.M., Coetzee T., Comi G., Correale J., Fazekas F., Filippi M., Freedman M.S., Fujihara K., Galetta S.L., Hartung H.P., Kappos L., Lublin F.D., Marrie R.A., Miller A.E., Miller D.H., Montalban X., Mowry E.M., Sorensen P.S., Tintoré M., Traboulsee A.L., Trojano M., Uitdehaag B.M.J., Vukusic S., Waubant E., Weinshenker B.G., Reingold S.C., Cohen J.A. (2018). Diagnosis of multiple sclerosis: 2017 revisions of the McDonald criteria.. Lancet Neurol..

[14] (2025). Introductory Guide MedDRA Version 24.1.. https://alt.meddra.org/files_acrobat/intguide_24_1_English.pdf.

[15] Gudesblatt M., Bumstead B., Buhse M., Zarif M., Morrow S.A., Nicholas J.A., Hancock L.M., Wilken J., Weller J., Scott N., Gocke A., Lewin J.B., Kaczmarek O., Mendoza J.P., Golan D. (2024). De-escalation of disease-modifying therapy for people with multiple sclerosis due to safety considerations: Characterizing 1-year outcomes in 25 people who switched from ocrelizumab to diroximel fumarate.. Adv. Ther..

[16] Selmaj K., Hartung H.P., Mycko M.P., Selmaj I., Cross A.H. (2024). MS treatment de-escalation: Review and commentary.. J. Neurol..

[17] Cotchett K.R., Dittel B.N., Obeidat A.Z. (2021). Comparison of the efficacy and safety of Anti-CD20 B cells depleting drugs in multiple sclerosis.. Mult. Scler. Relat. Disord..

[18] Wang M., Otto C., Fernández Zapata C., Dehlinger A., Gallaccio G., Diekmann L.M., Niederschweiberer M., Schindler P., Körtvélyessy P., Kunkel D., Paul F., Ruprecht K., Böttcher C. (2025). Comprehensive analysis of B cell repopulation in ocrelizumab-treated patients with multiple sclerosis by mass cytometry and proteomics.. iScience.

[19] Hauser S.L., Kappos L., Arnold D.L., Bar-Or A., Brochet B., Naismith R.T., Traboulsee A., Wolinsky J.S., Belachew S., Koendgen H., Levesque V., Manfrini M., Model F., Hubeaux S., Mehta L., Montalban X. (2020). Five years of ocrelizumab in relapsing multiple sclerosis.. Neurology.

[20] Boudot de la Motte M., Louapre C., Papeix C., Depaz R., Assouad R., Roux T., Lubetzki C., Maillart E. (2021). Challenges of switching towards anti-CD20 monoclonal antibodies in RR-MS: A monocentric study.. Mult. Scler. Relat. Disord..

[21] Maggi P., Bulcke C.V., Pedrini E., Bugli C., Sellimi A., Wynen M., Stölting A., Mullins W.A., Kalaitzidis G., Lolli V., Perrotta G., El Sankari S., Duprez T., Li X., Calabresi P.A., van Pesch V., Reich D.S., Absinta M. (2023). B cell depletion therapy does not resolve chronic active multiple sclerosis lesions.. EBioMedicine.

[22] Bar-Or A., Thanei G.A., Harp C., Bernasconi C., Bonati U., Cross A.H., Fischer S., Gaetano L., Hauser S.L., Hendricks R., Kappos L., Kuhle J., Leppert D., Model F., Sauter A., Koendgen H., Jia X., Herman A.E. (2023). Blood neurofilament light levels predict non-relapsing progression following anti-CD20 therapy in relapsing and primary progressive multiple sclerosis: Findings from the ocrelizumab randomised, double-blind phase 3 clinical trials.. EBioMedicine.

[23] D’Amico E., Zanghì A., Leone C., Tumani H., Patti F. (2016). Treatment-related progressive multifocal leukoencephalopathy in multiple sclerosis: A comprehensive review of current evidence and future needs.. Drug Saf..

[24] Martin R. (2018). Understanding risk of PML through multiple sclerosis.. Lancet Neurol..

[25] D’Amico E., Patti F., Zanghì A., Zappia M. (2016). A personalized approach in progressive multiple sclerosis: The current status of disease modifying therapies (DMTs) and future perspectives.. Int. J. Mol. Sci..

[26] Ganjgahi H., Häring D.A., Aarden P., Graham G., Sun Y., Gardiner S., Su W., Berge C., Bischof A., Fisher E., Gaetano L., Thoma S.P., Kieseier B.C., Nichols T.E., Thompson A.J., Montalban X., Lublin F.D., Kappos L., Arnold D.L., Bermel R.A., Wiendl H., Holmes C.C. (2025). AI-driven reclassification of multiple sclerosis progression.. Nat. Med..

[27] Smets I., Versteegh M., Huygens S., Corsten C., Wokke B., Smolders J. (2023). Health-economic benefits of anti-CD20 treatments in relapsing multiple sclerosis estimated using a treatment-sequence model.. Mult Scler. J. Exp. Transl. Clin..

[28] Soelberg Sorensen P., Giovannoni G., Montalban X., Thalheim C., Zaratin P., Comi G. (2019). The multiple sclerosis care unit.. Mult. Scler..

